# Relationship Between Health Literacy, Quality of Life, and Treatment Adherence in Patients with Acute Coronary Syndrome

**DOI:** 10.3928/24748307-20230320-01

**Published:** 2023-06

**Authors:** Mohammad Ali Zakeri, Asghar Tavan, Ali Esmaeili Nadimi, Golamreza Bazmandegan, Maryam Zakeri, Nadia Sedri

## Abstract

**Background:**

Acute coronary syndrome is a significant global health concern that can affect patients' health outcomes and quality of life. In addition, adherence to treatment and health literacy can affect health outcomes.

**Objective:**

This study aimed to investigate the relationship between treatment adherence, health literacy, and quality of life among patients with acute coronary syndrome.

**Methods:**

This cross-sectional study was conducted on 407 patients in Iran from April 2019 to November 2019. Patients were selected by convenience sampling method. Data were collected using demographic questionnaire, World Health Organization Quality of Life Brief Version, Adherence to Treatment Questionnaire, and Health Literacy for Iranian Adults questionnaire. SPSS 25 was used for statistical analysis.

**Results:**

Based on descriptive statistics in this study, most of the participants had good treatment adherence level (56.5%); 28.7% of the participants had insufficient health literacy level. The mean score of quality of life was 51.41 ± 12.03, which was greater than the midpoint of the questionnaire. Furthermore, Pearson's correlation coefficient showed a negative association between health literacy, treatment adherence (r = −0.167, *p* < .01), and quality of life (r = −0.153, *p* < .01), and a positive association between treatment adherence and quality of life (*r* = 0.169, *p* < .01).

**Conclusion:**

The results of the current study showed a negative relationship between health literacy, quality of life, and treatment adherence among patients with acute coronary syndrome. [***HLRP: Health Literacy Research and Practice*. 2023;7(2):e71–e79.**]

## Abstract (plain language summary)

**Plain Language Summary: **We investigated the relationship between treatment adherence, health literacy, and quality of life among patients with acute coronary syndrome. Most of the participants had good treatment adherence level; 28.7% of the participants had insufficient health literacy level. The results of the study showed a negative relationship between health literacy, quality of life, and treatment adherence among patients with acute coronary syndrome.

Rapid economic and social changes in recent decades combined with a lack of public knowledge in many Mediterranean and Middle Eastern countries, including Iran, (Malekzadeh et al., 2016), have created significant health problems for patients with common cardiovascular disease (CVD) (Sadeghi et al., 2021). One of the most important CVDs is acute coronary syndrome (ACS), which includes unstable angina, ST-elevation myocardial infarction, and non-ST elevation myocardial infarction (Soltani et al., 2016). Some studies have shown that most patients with ACS do not have adequate information about their medical condition, which can affect their treatment adherence (Fors et al., 2014).

Adherence to prescribed medications, fluid limitations, dietary restrictions (Nabolsi et al., 2015), and changes in lifestyle advised by health care providers are all examples of patient treatment adherence (Takaki & Yano, 2006). Some studies defined adherence to treatment in patients with ACS as adherence to diet, lifestyle, physical activity, and economic status (Notara et al., 2016). Patients with ACS are at risk for serious medication errors (SME), which are a serious part of adherence to treatment. Furthermore, cardiovascular drugs commonly account for 14% of adverse drug events after discharge (Schnipper et al., 2010) due to treatment nonadherence (Jencks et al., 2009). Therefore, patients with ACS are more likely to be hospitalized, and their medical costs increase.

On the other hand, treatment nonadherence in patients with ACS can have a psychological effect on their quality of life (QOL). Social and economic problems and aging are factors that reduce the QOL of patients with ACS. There has been little research on the prevention of depression in patients with ACS, which is an important factor in their QOL and treatment adherence (Notara et al., 2016). There are secondary prevention programs aimed at improving the QOL of patients with ACS by providing appropriate education and raising their level of health literacy (HL) because patients with ACS require prolonged care and health information to control the disease and improve their health status (Evangelista et al., 2010).

HL is one's ability to access, analyze, and accept health care information to make decisions about wellbeing (Masoompour et al., 2017). HL helps patients find enough information about their illness to make the right decision about it (Mavreles Ogrodnick et al., 2021). Different studies have shown that low level of HL is influenced by social, economic, and political factors (Schillinger et al., 2022) and postpones early disease diagnosis, causes inability to gain self-care skills (Lee et al., 2010), increases use of emergency services, hospital stay, disease prevalence, and eventually mortality (Afshari et al., 2014). Patients with a low level of HL may represent SME due to poor treatment adherence to drugs (Kripalani et al., 2008). Low HL is also seen in high-income countries (González-Chica et al., 2016). Limited research investigated the effects of HL on QOL of the patients with CVD (Macabasco-O'Connell et al., 2011). Scientific evidence indicates that insufficient HL has an effect on QOL costs (Nesbitt et al., 2014).

There appears to be a gap in the health care of patients with ACS, and these patients have a high mortality rate. Based on different studies, patients with low education, low income, living in rural and sparsely populated areas, women and older adults do not have adequate health literacy. A recent study was conducted to investigate the relationship between HL, treatment adherence, and QOL in patients with ACS.

## Method

### Data Source and Sample

This descriptive-correlational study was conducted in Ali-Ebne-Abitaleb Hospital affiliated to Rafsanjan University of Medical Sciences from April 2019 to November 2019. The research sample was patients with ACS in cardiac care unit (CCU) of the hospital. The inclusion criteria were patients age 20 to 80 years (Malekzadeh et al., 2016) with ACS in CCU, who took the cardiac medications and did not have mental illnesses. The exclusion criteria included the patient's unwillingness to continue cooperation and incomplete questionnaires. The sample size was 407 individuals by using the study of Qobadi (2015), the alpha of 95%, power of 90%, and *r* = 0.19, (Qobadi et al., 2015). Sampling continued until the sample size reached 407 people. For this purpose, 430 questionnaires were distributed, 23 of which were excluded due to incomplete questionnaires or patient's withdrawal from the study. The researchers began their work after acquiring the code of ethics No. IR.RUMS.REC. 1397.212 from the Ethics Committee of Kerman University of Medical Sciences. Using a convenient and simple method, the researchers selected the eligible participants from the list of the patients in the CCU. After the research method was explained, the participants signed the consent form and filled the self-report questionnaires. Percentage, frequency, mean, and standard deviation were used to determine the sample characteristics. Pearson correlation coefficient test and multivariate linear regression test were used. SPSS 25 was used for statistical analysis. The data collection tools included Health Literacy for Iranian Adults (HELIA), the QOL questionnaire developed by the World Health Organization Quality of Life Brief Version (WHOQOL-BREF), Adherence to Treatment Questionnaire (ATQ), and demographic information.

### Health Literacy for Iranian Adults

Montazeri et al. (2014) tested the validity and reliability of the questionnaire in Iran with Cronbach's alpha coefficient ranging from 0.72 to 0.89. The subscales of this questionnaire included reading (4 items, scores of 4–20), access (6 items, scores of 6–30), understanding (7 items, scores of 7–35), appraisal (4 items, scores of 4–20), and decision-making and behavior (12 items, scores of 12–60). Total score of the items related to these five subscales was 33, with a minimum of 33 and maximum of 165. The scores were assigned to four categories: *insufficient* (0–50), *fairly enough* (50.1-66), *sufficient* (66.1-84), and *excellent* (84.1-100) (Montazeri et al., 2014). In this study, the reliability of the HELIA scale was 0.95 using Cronbach's alpha coefficient.

### World Health Organization Quality of Life Brief Version

The WHOQOL-BREF questionnaire was developed by the World Health Organization in collaboration with 15 international centers in 1989. This questionnaire consists of 26 items with four domains: psychological, physical health, environmental, social relationship. Each item was scored on a scale of 1 to 5, with scores ranging from *never* to *quite high;* or *very dissatisfied*, *dissatisfied*, *relatively dissatisfied*, *satisfied*, *very satisfied*; or *never*, *rarely*, *sometimes*, *many times*, *always*). Nejat et al. (2006) tested the validity and reliability of the questionnaire in Iran. The Cronbach's alpha coefficients were 0.77, 0.77, 0.75, and 0.84 for physical health, mental health, social relations and environmental health, respectively, and total Cronbach's alpha coefficient was 0.78 (41). All coefficients were significant at the level of 0.01 (Nejat et al., 2006). In this study, the reliability of the QOL scale was 0.88 using Cronbach's alpha coefficient.

### Adherence to Treatment Questionnaire

The fourth part of the questionnaire included adherence to treatment on a 6-point Likert scale ranging from *completely* (five) to *never* (zero). This questionnaire had seven sub-scales: focus on treatment (0–45), willingness to participate in treatment (0–35), ability to adapt (0–35), adapting the treatment with life plan (0–25), adherence to treatment (0–20), commitment to treatment (0–25), and uncertainty in the implementation of treatment (0–15). The percentage of patients adhering to treatment was calculated using the following criteria: *very good* (75–100 percent), *good* (50–74 percent), *medium* (26–49 percent), and *weak* (0–25 percent). Face validity was determined qualitatively with the participation of 10 patients and 10 specialists, and content validity was determined qualitatively and quantitatively with the participation of 15 patients and 26 specialists. The mean content validity index of the questionnaire was 0.914. The questionnaire's internal consistency was calculated by using Cronbach's alpha (*α* = 0.921) and the questionnaire's reliability was stable by performing a re-test after 2 weeks (*r* = 0.875) (Seyed Fatemi et al., 2018). In this study, the reliability of the ATQ scale was 0.94 using Cronbach's alpha coefficient.

### Demographic Information

The demographic questionnaire consisted of age, sex, marital status, income, level of education, type of insurance, smoking and addiction, family history, symptoms associated with the disease and drugs used.

## Results

### General Characteristics, Health literacy, Treatment Adherence, and Quality of Life

**Table 1** shows that the mean age was 61.61 ± 12.25 years (minimum = 21 and maximum = 80). Most of the participants were male, married, and unemployed, and did not have a high school diploma, with no history of hospital stay and other illnesses. Pearson correlation coefficient showed a significant positive association between age, HL, and QOL (*p* < .001). QOL was correlated with the patients' gender (*p* = .011), marital status (*p* = .021), history of hospital stays (*p* < .001), diagnosis (*p* = .004), and other illnesses (*p* = .009). HL was associated with the participants' gender (*p* = .004), level of education (*p* < .001), employment status (*p* = .001), income (*p* < .001), and other illnesses (*p* = .025). Treatment adherence was associated with participants' gender (*p* = .012), diagnosis (*p* = .005), level of education (*p* < .001), and other illnesses (*p* = .032).

**Table 1:**
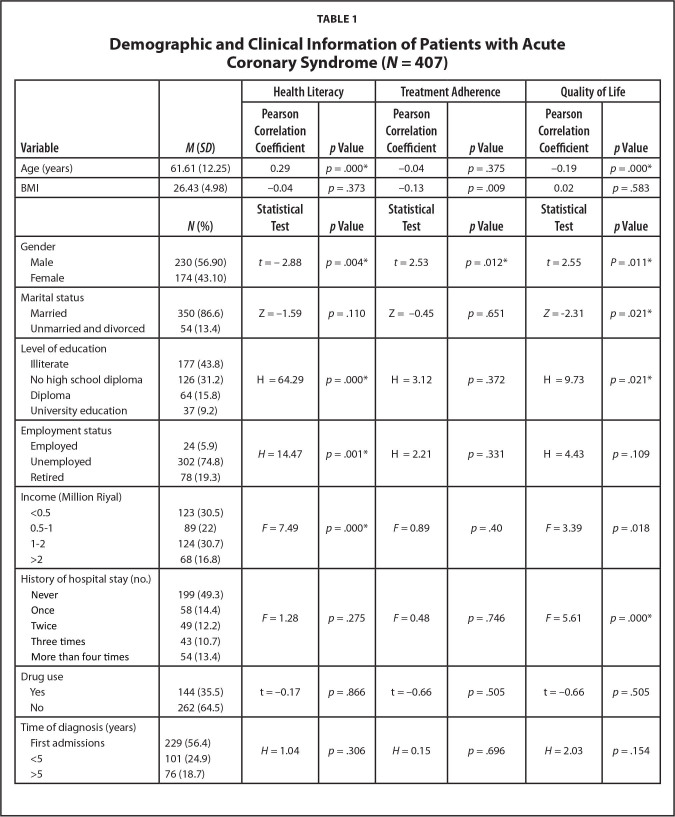

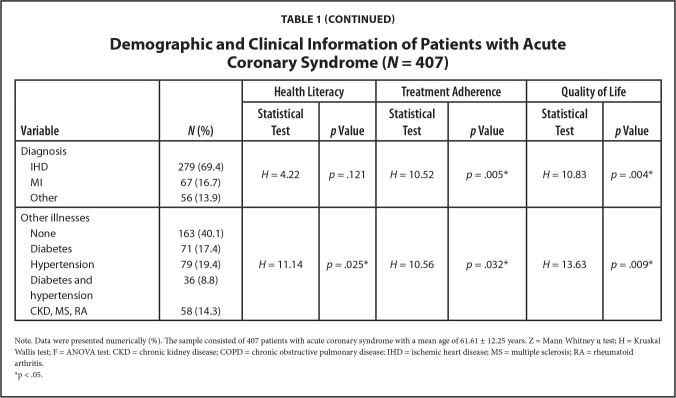
Demographic and Clinical Information of Patients with Acute Coronary Syndrome (*N* = 407)

**Variable**	***M* (*SD*)**	**Health Literacy**		**Treatment Adherence**		**Quality of Life**	

		**Pearson Correlation Coefficient**	***p* Value**	**Pearson Correlation Coefficient**	***p* Value**	**Pearson Correlation Coefficient**	***p* Value**

Age (years)	61.61 (12.25)	0.29	*p* = .000^*^	−0.04	*p* = .375	−0.19	*p* = .000^*^

BMI	26.43 (4.98)	−0.04	*p* = .373	−0.13	*p* = .009	0.02	*p* = .583

	***N* (%)**	**Statistical Test**	***p* Value**	**Statistical Test**	***p* Value**	**Statistical Test**	***p* Value**

Gender							
Male	230 (56.90)	*t* = − 2.88	*p* = .004^*^	*t* = 2.53	*p* = .012^*^	*t* = 2.55	*P* = .011^*^
Female	174 (43.10)						

Marital status							
Married	350 (86.6)	Z = −1.59	*p* = .110	Z = −0.45	*p* = .651	*Z* = −2.31	*p* = .021^*^
Unmarried and divorced	54 (13.4)						

Level of education							
Illiterate	177 (43.8)	H = 64.29	*p* = .000^*^	H = 3.12	*p* = .372	H = 9.73	*p* = .021^*^
No high school diploma	126 (31.2)						
Diploma	64 (15.8)						
University education	37 (9.2)						

Employment status							
Employed	24 (5.9)	*H* = 14.47	*p* = .001^*^	H = 2.21	*p* = .331	H = 4.43	*p* = .109
Unemployed	302 (74.8)						
Retired	78 (19.3)						

Income (Million Riyal)							
<0.5	123 (30.5)	*F* = 7.49	*p* = .000^*^	*F* = 0.89	*p* = .40	*F* = 3.39	*p* = .018
0.5–1	89 (22)						
1–2	124 (30.7)						
>2	68 (16.8)						

History of hospital stay (no.)							
Never	199 (49.3)	*F* = 1.28	*p* = .275	*F* = 0.48	*p* = .746	*F* = 5.61	*p* = .000^*^
Once	58 (14.4)						

Twice	49 (12.2)						
Three times	43 (10.7)						
More than four times	54 (13.4)						

Drug use							
Yes	144 (35.5)	t = −0.17	*p* = .866	t = −0.66	*p* = .505	t = −0.66	*p* = .505
No	262 (64.5)						

Time of diagnosis (years)							
First admissions	229 (56.4)	*H* = 1.04	*p* = .306	*H* = 0.15	*p* = .696	*H* = 2.03	*p* = .154
<5	101 (24.9)						
>5	76 (18.7)						

Diagnosis							
IHD	279 (69.4)	*H* = 4.22	*p* = .121	*H* = 10.52	*p* = .005^*^	*H* = 10.83	*p* = .004^*^
MI	67 (16.7)						
Other	56 (13.9)						

Other illnesses							
None	163 (40.1)	*H* = 11.14	*p* = .025^*^	*H* = 10.56	*p* = .032^*^	*H* = 13.63	*p* = .009^*^
Diabetes	71 (17.4)						
Hypertension	79 (19.4)						
Diabetes and hypertension	36 (8.8)						
CKD, MS, RA	58 (14.3)						

Note. Data were presented numerically (%). The sample consisted of 407 patients with acute coronary syndrome with a mean age of 61.61 ± 12.25 years. Z = Mann Whitney u test; H = Kruskal Wallis test; F = ANOVA test. CKD = chronic kidney disease; COPD = chronic obstructive pulmonary disease; IHD = ischemic heart disease; MS = multiple sclerosis; RA = rheumatoid arthritis.

* p < .05.

In **Table 2**, the mean score of treatment adherence was 117.05 ± 28.36, which was greater than the midpoint of the questionnaire (score = 100). The highest mean value was found in focus on treatment (26.04 ± 8.07), while the lowest mean value was found in doubt about the implementation of treatment (9.48 ±  3.70) (**Table 2**). Treatment adherence level was *very good* in 56 (13.8%), *good* in 230 (56.5%), *median* in 113 (27.8%), and *weak* in one participant (0.2%). Most of the participants adhered to their treatment well (56.5%). In addition, **Table 2** showed that the mean score of HL was 101.10 ± 30.95, which was greater than the midpoint of the questionnaire (score = 85). The mean scores of HL variables were between 13.09 and 34.28. HL levels were excellent in 72 (17.7%), enough in 96 (23.6%), neither sufficient nor insufficient in 107 (26.3%), and insufficient in 117 participants (28.7%). Finally, the mean score of QOL was 51.41 ± 12.03, which was greater than the midpoint of the questionnaire (score = 50).

**Table 2:**
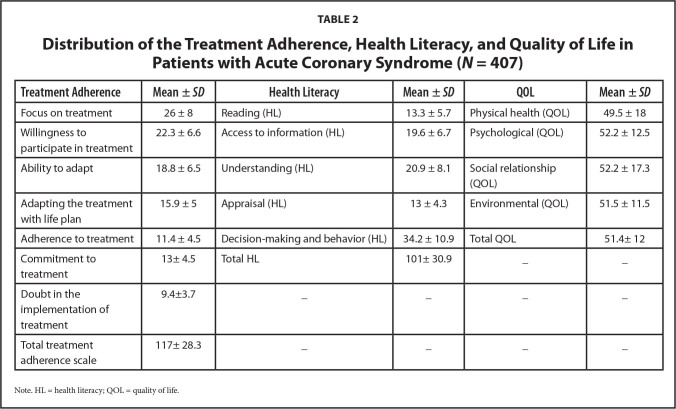
Distribution of the Treatment Adherence, Health Literacy, and Quality of Life in Patients with Acute Coronary Syndrome (*N* = 407)

**Treatment Adherence**	**Mean ± *SD***	**Health Literacy**	**Mean ± *SD***	**QOL**	**Mean ± *SD***
Focus on treatment	26 ± 8	Reading (HL)	13.3 ± 5.7	Physical health (QOL)	49.5 ± 18
Willingness to participate in treatment	22.3 ± 6.6	Access to information (HL)	19.6 ± 6.7	Psychological (QOL)	52.2 ± 12.5
Ability to adapt	18.8 ± 6.5	Understanding (HL)	20.9 ± 8.1	Social relationship (QOL)	52.2 ± 17.3
Adapting the treatment with life plan	15.9 ± 5	Appraisal (HL)	13 ± 4.3	Environmental (QOL)	51.5 ± 11.5
Adherence to treatment	11.4 ± 4.5	Decision-making and behavior (HL)	34.2 ± 10.9	Total QOL	51.4± 12
Commitment to treatment	13± 4.5	Total HL	101± 30.9	_	_
Doubt in the implementation of treatment	9.4±3.7	_	_	_	_
Total treatment adherence scale	117± 28.3	_	_	_	_

Note. HL = health literacy; QOL = quality of life.

**Table 3** indicated Pearson correlation coefficient between HL, QOL, and treatment adherence. A significant association between HL, treatment adherence (r = −0.167, *p* < .01), and QOL was observed (r = −0.153, *p* < .01). There was a significant positive correlation between treatment adherence, and QOL (*r* = 0.169, *p* < .01).

**Table 3:**
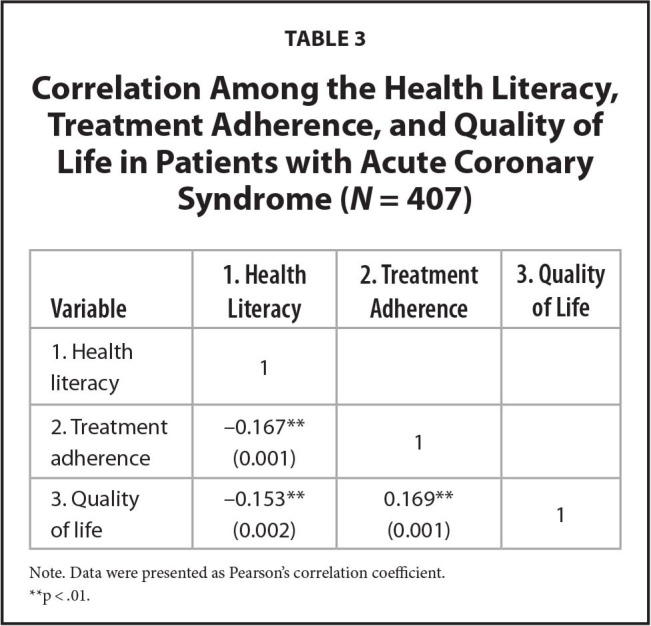
Correlation Among the Health Literacy, Treatment Adherence, and Quality of Life in Patients with Acute Coronary Syndrome (*N* = 407)

**Variable**	**1. Health Literacy**	**2. Treatment Adherence**	**3. Quality of Life**
1. Health literacy	1		
2. Treatment adherence	−0.167^**^ (0.001)	1	
3. Quality of life	−0.153^**^ (0.002)	0.169^**^ (0.001)	1

Note. Data were presented as Pearson's correlation coefficient.

** p <  .01.

### Regression Analysis

***Variables correlated with health literacy, quality of life, and treatment adherence.*** **Table 4** showed the results of multivariate linear regression with backward method to determine the significant correlation between variables, HL, treatment adherence, and QOL in the participants. Seventeen percent of the variance of HL was predicted by level of education, employment status, treatment adherence, and income (R^2^ = 17%), and the best predictor was education level (*p* < .001). HL, QOL, diagnosis, and other illnesses predicted 7 % of the variance of treatment adherence (R^2^ = 7 %), and the best predictor was HL (*p* < .001). Finally, treatment adherence, level of education, and history of hospital stay predicted 8 % of the variance of QOL (R^2^ = 8 %) and based on β, the best predictor was history of hospital stay (*p* < .001) (**Table 4**).

**Table 4:**
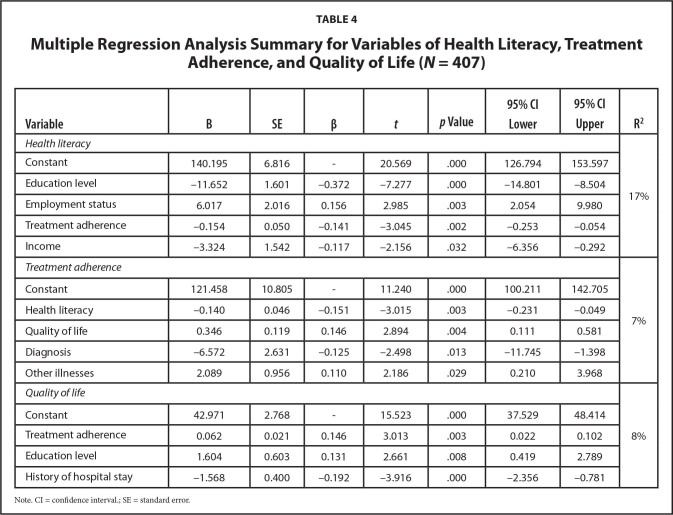
Multiple Regression Analysis Summary for Variables of Health Literacy, Treatment Adherence, and Quality of Life (*N* = 407)

**Variable**	**B**	**SE**	**β**	***t***	***p* Value**	**95% CI Lower**	**95% CI Upper**	***R* ^2^**
*Health literacy*								
Constant	140.195	6.816	-	20.569	.000	126.794	153.597	17%
Education level	−11.652	1.601	−0.372	−7.277	.000	−14.801	−8.504	
Employment status	6.017	2.016	0.156	2.985	.003	2.054	9.980	
Treatment adherence	−0.154	0.050	−0.141	−3.045	.002	−0.253	−0.054	
Income	−3.324	1.542	−0.117	−2.156	.032	−6.356	−0.292	
*Treatment adherence*								
Constant	121.458	10.805	-	11.240	.000	100.211	142.705	7%
Health literacy	−0.140	0.046	−0.151	−3.015	.003	−0.231	−0.049	
Quality of life	0.346	0.119	0.146	2.894	.004	0.111	0.581	
Diagnosis	−6.572	2.631	−0.125	−2.498	.013	−11.745	−1.398	
Other illnesses	2.089	0.956	0.110	2.186	.029	0.210	3.968	
*Quality of life*								
Constant	42.971	2.768	-	15.523	.000	37.529	48.414	8%
Treatment adherence	0.062	0.021	0.146	3.013	.003	0.022	0.102	
Education level	1.604	0.603	0.131	2.661	.008	0.419	2.789	
History of hospital stay	−1.568	0.400	−0.192	−3.916	.000	−2.356	−0.781	

Note. CI = confidence interval.; SE = standard error.

## Discussion

The results of the current study showed a negative relationship between HL, QOL, and treatment adherence in patients with ACS. According to findings, most of the participants had *good* treatment adherence, while only 17% of them had *excellent* HL, and 28.8% of them had *insufficient* HL. Some studies, which were consistent with results of the present study, showed that patients with high HL had low treatment adherence (Bauer et al., 2013; Loke et al., 2012; Ostini & Kairuz, 2014; Waite et al., 2008; Waldrop-Valverde et al., 2009). Unlike our research (Coskun & Bagcivan, 2021; Zhang et al., 2014), indicated a significant association between HL and treatment adherence in patients. They reported the effect of HL on treatment adherence.

The present study showed a negative relationship between HL and treatment adherence. Some studies could not find a significant relationship between treatment adherence and health literacy. This relationship may be positive in some studies and negative in others. Also, they did not show a predictable relationship between HL and some aspects of treatment adherence (Ostini & Kairuz, 2014; Pignone & DeWalt, 2006). The relationship between HL and treatment adherence was specific to ACS patients in the recent study, which can have different outcomes in other patients. Studies on the relationship between HL and treatment adherence indicate that the relationship between disease, the HL, and treatment adherence is not sufficiently strong. However, research suggested that the relationship between HL and treatment adherence was less evident (Keller et al., 2008).

The results of multivariate linear regression test showed that diagnosis, other illnesses, QOL, and HL were the best predictors of treatment adherence. Most of the study participants were ischemic heart disease and had other illnesses (chronic disease), such as diabetes, hypertension, and chronic diseases. Studies reported that other illnesses, such as diabetes and hypertension were predictors of treatment adherence, which was in line with the results of the present study (Faint et al., 2017; Loke et al., 2012; Miller, 2016). It seems that treatment adherence is higher in patients with ACS and other illnesses because their symptoms appear earlier.

The multivariate linear regression test also showed that level of education, employment status, treatment adherence, and income predicted HL in patients with ACS. Some studies supported this result, showing that the level of education could increase the amount of HL (Chen et al., 2020; Mackenbach et al., 2008; van Der Heide et al., 2013). Nevertheless, some studies showed that level of education was not an adequate predictor of HL because some individuals with higher education had low HL (Adams et al., 2013). In addition, some people with low levels of education can develop health literacy skills (Sørensen et al., 2012). The results of this study also highlighted a negative relationship between HL and QOL in patients with ACS. Although QOL and HL are essential in health care, their relationship is unknown in some studies, particularly among chronically ill patients, who frequently use healthcare services. Some studies found both negative and positive relationships between health literacy and some aspects of QOL. Couture et al. (2017) showed no correlation between HL and some aspects of QOL among regular users of medical services. A study demonstrated a significant association between HL, psychological, and physical aspects of QOL (Kooshyar et al., 2014), which was consistent with the study of Murray et al. (2009) on patients with heart failure but inconsistent with the present study. The present study did not show the positive effect of HL on QOL, which might be due to various cultural, social factors, and media. This issue can be investigated in future studies.

This study also discussed about the predictors of QOL in patients with ACS. According to multivariate linear regression, hospital stay, treatment adherence, and educational level were predictors of QOL in patients with ACS. Some research supported the results of this study (Kweon et al., 2017; Nesbitt et al., 2014; Oliveira et al., 2016). Oliveira et al. (2016), found that long hospital stay could increase the risk of morbidity and mortality, and influenced the patient's QOL. Prolonged hospital stay seems to be a sign of chronic and serious disease; therefore, it increases the patient's dependence on various treatments and affects their QOL. Kweon et al. (2017), discovered that low educational level was associated with poor QOL. Nevertheless, there is contradictory results in the literature about the level of education and QOL. Park et al. (2018), found that the level of education was not related to QOL, even though it was directly related to other problems, such as chronic disease (Park et al., 2018). In addition, some studies showed that income and employment status could have a greater impact on improving people's QOL than the level of education (González-Chica et al., 2016).

Finally, treatment adherence can motivate people to use health-related data and guidance, then medical staff should pay much more attention to patients with ACS to get understandable information. It is important to early detect poor treatment adherence in patients with ACS, try to change their lifestyles, and perform motivational education services to improve their treatment adherence.

This study also had limitations. This is a cross-sectional study and different results may be seen in different time periods. Some patients filled in the questionnaires incompletely, who were excluded from the study, and sampling was continued to reach the specified sample size.

## Conclusion

The findings of this study highlighted a negative relationship between HL, treatment adherence, and QOL in patients with ACS. Our results suggested that treatment adherence could improve the QOL of patients with ACS. As a result, it is suggested that patients with ACS receive the appropriate training to improve their treatment adherence. Furthermore, the results demonstrated a relationship between some demographic and socioeconomic variables and the main variables investigated in this study.
